# Oxidative stress in periodontitis and the application of antioxidants in treatment: a narrative review

**DOI:** 10.3389/fphys.2025.1485367

**Published:** 2025-05-13

**Authors:** Wei Liu, Daoyu Guo

**Affiliations:** ^1^ School of Clinical Medicine, Qilu Medical University, Zibo, China; ^2^ School of Medicine and Nursing, Huzhou University, Huzhou, China

**Keywords:** oxidative stress, periodontitis, antioxidants, inflammation, tissue repair

## Abstract

Periodontitis has turned into a general oral disease defined by chronic inflammation of the gums and helping tissues of the teeth. It dramatically influences both oral and systemic health and is a main trigger of tooth loss. Periodontitis is tightly linked to oxidative stress, and evidence reveals the utilization of certain antioxidants in related treatments. Our review delves into literature in periodontitis, centering on the latest therapeutic strategies and mechanistic evidences. This review starts by depicting the epidemiological features and pathogenic mechanisms of periodontitis, highlighting the vital mechanism of microbial community modefications, host immune responses, and genetic factors in disease development. Existing treatment approaches for periodontitis involve mechanical cleaning, antibiotic therapy, surgical interventions, and pharmacological treatments, with a comprehensive exploration on the strengths and limitations of each strategy and their related clinical utilizations. Besides, this review investigates emerging therapies, like anti-inflammatory treatments, immune modulation therapies, and biotechnological utilization in the control of periodontitis. It particularly stresses the protective effects of antioxidants and their mechanistic researches in periodontitis, highlighting their potential in slowing inflammatory responses and boosting tissue repair. Ultimately, our review intends to the concept of individual therapy and investigates future directions, comprising the utilization of precision medicine in periodontitis control and the individual design of treatment utilization.

## 1 Introduction

Periodontitis is defined as a chronic inflammatory disease mainly affecting the tissues which supports the teeth, including the gums, periodontal ligament, and alveolar bone. Generally speaking, it ranks among the most prevalent oral diseases, obviously impacting individual oral health and quality of life (Könönen et al., 2019). According to the World Dental Federation (FDI), severe periodontitis have an impact on nearly 19% of the adult population globally, making up for over one billion cases (Könönen et al., 2019). Besides, the prevalence of mild to moderate forms of periodontitis among adults around the world is about 50% (Könönen et al., 2019). Hence, periodontitis is regarded as a prevalent and serious global health issue impacting oral health.

Oxidative stress refers to the phenomenon where excessive production of oxygen free radicals and other oxidants in the cellular and extracellular environment overwhelms the body’s antioxidant defense systems, which leads to cellular functional damage (Wang et al., 2017a), (Liu et al., 2017). Oxidative stress is thought as a essential pathophysiological process in the onset of periodontitis, exacerbating inflammatory reactions and including the health of gums and surrounding tissues, thereby increasing disease severity (Wang et al., 2017a), (Liu et al., 2017).

In the management of periodontitis, antioxidant therapy has appeared as a potential treatment strategy garnering wide attention. By boosting oxidative stress status, antioxidants can alleviate clinical symptoms and disease progression. Antioxidants mitigate cellular component damage which is caused by oxidative stress, while playing a vital role in the treatment of diverse chronic inflammatory diseases (Blagov et al., 2024). Studies show that antioxidants neutralize reactive oxygen species (ROS), decrease cellular damage that is induced by oxidative stress, and as a consequense lower the severity of chronic inflammation. This therapeutic way extends beyond periodontitis to display possible efficacy in other chronic inflammatory diseases such as inflammatory bowel disease (IBD) (Rodrigues Junior et al., 2023). More clinical research and experiments in terms on antioxidant therapy, especially in deciding optimal drug dosages and treatment efficacy, stay pivotal for future investigations (Blagov et al., 2024). So through boosting endogenous antioxidant levels, antioxidant therapy provides a promising avenue not only for improving the level of oral health but also possibly providing new useful treatments for chronic inflammatory diseases such as periodontitis.

Above all, this review thoroughly summarizes the function of oxidative stress in periodontitis and discusses how antioxidants, by inhibiting oxidative stress, are applied in its treatment. Comparative analyses of these antioxidants' antioxidant influences and their therapeutic efficacy for periodontitis offer potential reference value for clinical treatments.

## 2 Oxidative stress and periodontitis

### 2.1 Basic concepts of oxidative stress

Oxidative stress refers to an imbalance between the production of reactive oxygen types (involving superoxide anions, hydrogen peroxide, etc.) and other oxidants in the cellular and extracellular environment, capable of damaging cell structures and functions (Albano et al., 2022). The generation of oxygen free radicals is mainly related to incomplete reduction processes in redox reactions, specifically under conditions of active cellular metabolism, inflammation, or external stress, where their production increases significantly (Albano et al., 2022), (Da Silva et al., 2023).

Under normal conditions, the body regulates and neutralizes these parlous oxidants by antioxidant systems (including superoxide dismutase, glutathione peroxidase, etc.), remaining redox balance within cells (Albano et al., 2022). But in inflammatory diseases such as periodontitis, the release of inflammatory mediators, local hypoxia, and activation of immune cells can obviously raise oxidative stress beyond the regulation ability of antioxidant systems, resulting in cellular damage and amplifying inflammation (Albano et al., 2022), (Menzel et al., 2021).

### 2.2 Relationship between oxidative stress and gingival tissue inflammation

Oxidative stress is very vital in the progress of periodontitis, tightly linked with inflammation of the gingival tissues. Periodontitis is an inflammatory disease of the gums and supporting tissues of the teeth lead by the addition of dental plaque (Tóthová and Celec, 2017). In the early stages of the disease, bacterial metabolic products in dental plaque can activate host cells to release inflammatory mediators like interleukin-1β, tumor necrosis factor-α, further contributing to the release of oxygen free radicals and other types of oxidants (Mazurek-Mochol et al., 2024).

To much production of oxidants results into oxidative damage to cells and matrix molecules in the gingival tissues, like lipid peroxidation, protein oxidation, and DNA damage, aggravating the severity and duration of the inflammatory response (Chapple and Matthews, 2000). In addition, oxidative stress can activate apoptosis in fibroblasts and osteoblasts, stopping their differentiation and function, further intensifying damage and loss of periodontal tissues (Chapple and Matthews, 2000).

### 2.3 Role of oxidative stress in the pathogenesis of periodontitis

**FIGURE 1:**
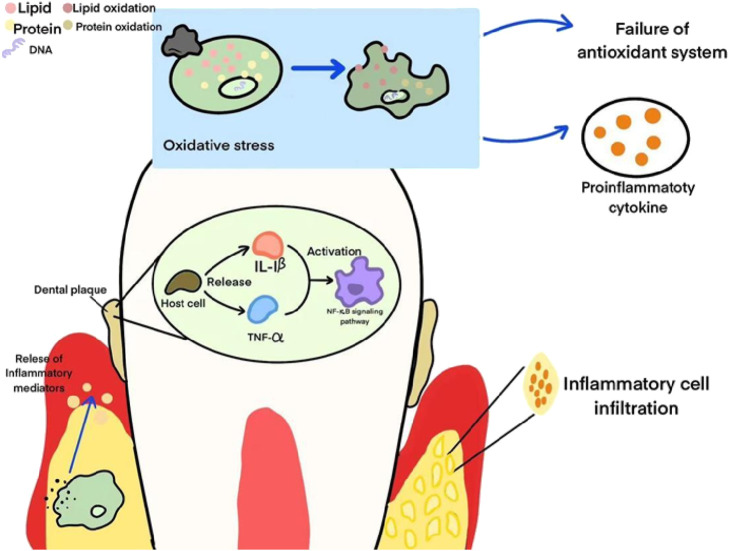
The role of oxidative stress in the pathogenesis of periodontitis. This figure illustrates the biological mechanisms associated with oral inflammation. First, oxidative stress leads to the oxidation of lipids and proteins, triggering the failure of the antioxidant system and the production of pro-inflammatory cytokines. At the same time, plaque stimulates host cells to release inflammatory mediators such as IL-1β and TNF-α, activates the NF-κB signaling pathway, and then recruits inflammatory cell infiltrates, ultimately triggering an inflammatory response.

In the pathogenesis of periodontitis, oxidative stress is of great importance. In the early stage, bacterial metabolic products motivate host cells to release various inflammatory mediators like as IL-1β and TNF-α, elevating local oxidative stress levels and increasing the generation of oxygen free radicals by multiple pathways. These oxygen free radicals lead to lipid, protein, and DNA damage inside cells, which leads to structural and functional abnormalities in periodontal tissues and escalates disease progression. Constant oxidative stress depletes the antioxidant system, promoting inflammation and tissue damage, then improving the advancement of periodontitis. Meanwhile, oxidative stress also contributes to the continuity and severity of inflammatory responses, thereby speeding up pathological tissue damage and aggravateing the development of periodontal diseases. Figure 1 shows the role of oxidative stress in the pathogenesis of periodontitis.

#### 2.3.1 Release of inflammatory mediators and increased oxidative stress

In the initial stages of periodontitis, bacterial metabolic products in dental plaque motivate host cells, resulting into the unleashing of various inflammatory mediators. These mediators comprise interleukin-1β (IL-1β), tumor necrosis factor-α (TNF-α), which shoulder vital responsibility in inflammatory responses (Han et al., 2023). Studies have displayed that the release of IL-1β and TNF-α not only intensifies local oxidative stress levels but also boosts the generation of oxygen free radicals through activating pathways like the NF-κB signaling pathway (Luo et al., 2024), (Zhu et al., 2022), (Yucel-Lindberg and Båge, 2013).

IL-1β, TNF-α, and other inflammatory mediators regulate the expression in a variety of cell types like alveolar bone osteoblasts, gingival fibroblasts, amplifying the damage of periodontal tissues and bone resorption processes (Yucel-Lindberg and Båge, 2013).

#### 2.3.2 Cellular dysfunction and apoptosis

In gingival tissues, oxidative stress causes lipid peroxidation, protein oxidation, and DNA damage. Lipid peroxidation mainly includes the reaction of ROS with lipid molecules in cell membranes, raising membrane fluidity and permeability, thereby having an impact on cell structure and function (Yadav, 2015). Protein oxidation causes odd protein structure and function, involving enzyme inactivation and distublance of cell signal transduction, straightly influencing the activity of fibroblasts and osteoblasts (Sharifi-Rad et al., 2020), (Martins et al., 2021).

Moreover, DNA damage activates stress response mechanisms within cells, possibly causing apoptosis or mutation, further compromising the health of gingival tissues (Martins et al., 2021). These molecular damages resulting from oxidative stress not only affect individual cells but may also affect the holistic structure and function of gingival tissues. Remaining cellular redox balance is important for avoiding such damages; proper antioxidant defense systems can help alleviate ROS-induced damage and keep normal physiological status of gingival tissues (Martins et al., 2021).

#### 2.3.3 Depletion of antioxidant systems

During the prolonged progression of chronic periodontitis, oxidative stress persists, causing depletion and imbalance of antioxidant systems (Shang et al., 2023). Periodontitis is featured by pathological dysbiosis of the microbial community within periodontal tissues, leading to chronic inflammation and structural damage to tooth-supporting tissues (Velsko and Warinner, 2025). This metabolic dysbiosis can cause the imbalance of antioxidant defense systems and apparently raising the risk of periodontal tissue destruction (Shang et al., 2023).

## 3 Antioxidants and their roles

### 3.1 Types and functions of main antioxidants

Antioxidants are compounds which is capable of neutralizing and clearing oxygen free radicals and other oxidants within cells, having a huge impact on the prevention and treatment of chronic inflammatory diseases. This chapter illustrates main types of antioxidants and their functional characteristics in the management of periodontitis. Table 1 demonstrates Functions, Actions, Clinical Applications and Therapeutic Effects of Antioxidant Substances in Periodontal Health.

**TABLE 1:** Functions, actions, clinical applications and therapeutic effects of antioxidant substances in periodontal health.

Antioxidant substances	Functions	Main actions and research findings	Clinical applications and therapeutic effects	References
Vitamin C (Ascorbic Acid)	As a water-soluble vitamin, vitamin C can obviously capture and neutralize various water-soluble oxygen free radicals, including hydroxyl radicals (•OH) and singlet oxygen (1O2), thereby reducing cellular damage caused by oxidative stress. Vitamin C can regenerate other antioxidants such as α-tocopherol (vitamin E), enhancing their antioxidant activity and helping to maintain oxidative balance within cells (Boo, 2022a; He et al., 2017).	Vitamin C neutralizes oral free radicals, reduces oxidative stress, and decreases release of gingival inflammatory mediators. Clinical trials show oral or topical vitamin C improves gum health, reducing inflammation symptoms like bleeding and swelling. Vitamin C promotes collagen synthesis and repair, crucial for periodontal tissue structure and function (Murererehe et al., 2022).	Reduces gingival bleeding and swelling, improves gingival health. Significant efficacy in non-surgical management of periodontitis (Fageeh et al., 2021a).	(Boo, 2022a), (He et al., 2017)
Vitamin E (α-Tocopherol)	Primary function in the cell membrane lipid layer, stabilizing and protecting cell membrane structure, particularly against lipid peroxides. By capturing and neutralizing free radicals, especially lipid peroxides such as lipid peroxides, it effectively prevents the continued propagation of chain reactions, maintaining the integrity of the cell membrane. Additionally, supplementation with vitamin E significantly increases the concentration of α-tocopherol in cell mitochondria and microsomes, further enhancing the protective effect of these subcellular structures against oxidative stress (Lauridsen and Jensen, 2012a; Galli et al., 2022), (Lauridsen and Jensen, 2012a; Galli et al., 2022).	Vitamin E, a fat-soluble antioxidant, protects cell membranes by inhibiting lipid peroxidation, crucial for reducing oxidative damage. Vitamin E supplementation lowers oxidative stress in periodontal tissues, alleviates inflammation, and supports periodontal health. Particularly effective in reducing inflammatory mediators in gingivitis and periodontitis treatments (Sardar et al., 1996).	Reduces periodontal pocket depth, improves clinical attachment level, benefits overall periodontal tissue health (Varela-López et al., 2018).	(Lauridsen and Jensen, 2012a); (Galli et al., 2022)
Glutathione	As the primary antioxidant inside cells, glutathione maintains oxidative balance by reacting with oxygen free radicals and other oxidants. Through NADPH-dependent glutathione reductase, oxidized glutathione (GSSG) is reduced back to its reduced form (GSH), helping to preserve the reducing environment within cells. This process also regenerates important antioxidants such as vitamin C and vitamin E, further enhancing cellular antioxidant capacity. Studies indicate that glutathione plays a crucial role in the onset and development of various diseases, including periodontitis. Reduced levels of glutathione in these diseases may increase cellular sensitivity to oxidative stress, thereby affecting cellular function (Kerksick and Willoughby, 2005; Bagatini et al., 2020), (Kerksick and Willoughby, 2005; Bagatini et al., 2020).	Glutathione, as a primary intracellular antioxidant, regulates cellular oxidative balance, protecting periodontal tissues. Lower glutathione levels in periodontitis correlate with increased gum bleeding and deeper periodontal pockets.3. Glutathione modifies protein S-glutathionylation, regulating cellular signaling pathways and responding to environmental changes (Ilkun and Boudina, 2013).	Improves antioxidant capacity, mitigates oxidative stress-induced tissue damage in periodontitis, broader systemic health benefits (Wang et al., 2017b).	(Kerksick and Willoughby, 2005); (Bagatini et al., 2020)
Carotenoids (β-Carotene, Lutein, etc.)	Carotenoids can effectively capture and neutralize oxygen free radicals such as singlet oxygen (1O2), thereby protecting periodontal cell membranes and organelles from oxidative damage. Their mechanism of action primarily involves their conjugated double bond structure, which enables carotenoids to undergo physical and chemical reactions with oxygen free radicals. Particularly under conditions of oxidative stress, this capability effectively preserves the integrity of cell membranes and organelle functions. Additionally, research indicates that carotenoids and their metabolites may transition between antioxidant and pro-oxidant roles depending on environmental factors such as oxygen concentration (Young and Lowe, 2018; Fiedor and Burda, 2014), (Young and Lowe, 2018; Fiedor and Burda, 2014).	Main Actions and Research Findings	Clinical Applications and Therapeutic Effects	(Young and Lowe, 2018); (Fiedor and Burda, 2014)

#### 3.1.1 Vitamin C (ascorbic acid)

Vitamin C is an fundamental water-soluble vitamin serving as a potential antioxidant in the human body. It effectively captures and neutralizes diverse water-soluble oxygen free radicals, comprising hydroxyl radicals (•OH) and singlet oxygen (1O2), leading to reduction of oxidative stress-induced cellular damage (Boo YC., 2022). Hydroxyl radicals are very reactive and can initiate lipid peroxidation, while vitamin C can neutralize them by donating electrons or protons, thereby stopping further oxidative damage (Naidu, 2003), (He et al., 2017). Besides, vitamin C can regenerate other antioxidants like α-tocopherol (vitamin E), promoting their antioxidant activity. This regeneration process benefits to maintain the oxidative balance within cells, heeping them from oxidative stress (He et al., 2017). Studies have dispalyed that vitamin C shows excellent antioxidant properties *in vivo*, especially in the processes of cardiovascular disease, cancer, and neurodegenerative diseases (Kalyanaraman, 2013). The antioxidant characteristics of vitamin C are not limited to direct neutralization of free radicals but also include complex interactions with other antioxidants, making it one of the crucial nutrients for keeping health and avoiding diseases (Kruk et al., 2022).

#### 3.1.2 Vitamin E (α-Tocopherol)

Vitamin E mainly serves in the cell membrane lipid layer to stabilize and protect the cell membrane structure from oxidative damage. Vitamin E lies dominantly in the phospholipid bilayer of the cell membrane, consisting of highly unsaturated fatty acids and is especially susceptible to oxidative damage (Lauridsen and Jensen, 2012a). By grabbing and neutralizing free radicals, in particular lipid peroxides like lipid peroxides, vitamin E can apparently stop the continued spread of chain reactions, thereby remaining the integrity of the cell membrane (Lauridsen and Jensen, 2012a), (Galli et al., 2022). Research has demonstrated that the α-tocopherol form of vitamin E is of great importance in the phospholipid bilayer of cell membranes, protecting the cell membrane not only by direct antioxidant action but also possibly by modulation of membrane-related bioactive molecules like enzymes and signaling molecules (Lauridsen and Jensen, 2012a). Moreover, supplementation with vitamin E has been displayed to obviously boost the concentration of α-tocopherol in cell mitochondria and microsomes, thereby improving the protective effect of these subcellular structures against oxidative stress (Lauridsen and Jensen, 2012a).

Hence, vitamin E, as a lipid-soluble antioxidant, is important in keeping cell structure and role by its localization and antioxidant mechanisms in the cell membrane.

#### 3.1.3 Glutathione

Glutathione (GSH), serving as the main antioxidant inside cells, is vital in maintaining oxidative balance within cells. It comprises of three amino acids: cysteine, glycine, and glutamic acid, which are able to react with oxygen free radicals and other oxidants to keep cells from oxidative damage (Kerksick and Willoughby, 2005; Poladian et al., 2023), (Kerksick and Willoughby, 2005; Poladian et al., 2023).

One of the major mechanisms of glutathione is to remain oxidative balance within cells through decreasing damaged cell components. Under oxidative stress, glutathione can change its oxidized form (GSSG) back to its reduced form (GSH) by the NADPH-dependent glutathione reductase. This process not only benefits to maintain the reducing environment within cells but also regenerates other crucial antioxidants like vitamin C and vitamin E, thereby boosting the cell’s antioxidant ability (Poladian et al., 2023). Studies have illustrated that glutathione is vital in the onset and development of various diseases, involving periodontitis. Reduced levels of glutathione in these diseases may improve the sensitivity of cells to oxidative stress, thereby damaging cellular functions (Bagatini et al., 2020).

Generally, glutathione, serving as the major antioxidant inside cells, not only directly erasing free radicals but also remains oxidative balance within cells by various mechanisms, which is crucial for keeping cellular health.

#### 3.1.4 Carotenoids (β-carotene, lutein, etc.)

Carotenoids are a series of potent antioxidants, primarily involvingβ-carotene, lutein, and others, with the ability to grab and neutralize oxygen free radicals like singlet oxygen (Young and Lowe, 2018). Their mechanism of action is mainly by their conjugated double bond structure, enabling carotenoids to effectively conduct physical and chemical reactions with oxygen free radicals, resulting into keepong the structure of periodontal cell membranes and organelles from oxidative damage (Young and Lowe, 2018). Studies have demonstrated that carotenoids, particularly β-carotene, lutein, and lycopene, showcase vital antioxidant capacity *in vivo* through quenching singlet oxygen (1O2) and other reactive oxygen species (ROS) by physical and chemical reactions (Young and Lowe, 2018), (Fiedor and Burda, 2014).

To be specific, carotenoids can efficiently neutralize parlous free radicals by physical quenching of singlet oxygen (1O2) and other ROS, which is importaant for protecting the integrity of cell membranes and organelle functions, particularly under conditions of oxidative stress (Fiedor and Burda, 2014). Additionally, research shows that carotenoids and their metabolites possibly transition between antioxidant and pro-oxidant roles relying on environmental causes like oxygen concentration (Machlin, 2019).

Hence, carotenoids, with their potential antioxidant features, is of great importance in keeping the structure and function of periodontal tissues from oxidative damage, emphasizing their possible application in keeping oral health.

### 3.2 Application research progress of antioxidants in the prevention and treatment of periodontitis

This section discusses the Application Research Progress of Antioxidants in the Prevention and Treatment of Periodontitis. Vitamin C, as a water-soluble antioxidant, is important in oral health through neutralising free radicals,d ecreasing oxidative pressure and hindering the release of Vitamin C, as a water-soluble antioxidant, is essential in oral health hrough neutralising free radicals, decreasing oxidative pressure and hindering the release of inflammatory mediators, particularly in decreasing gingival inflammation. Vitamin E, serving as a fat-soluble antioxidant, is helpful in the prevention and treatment of periodontal lesions through impeding lipid peroxidation, lessening inflammatory responses, and keeping cell membranes from free radical damage. Glutathione, as a major intracellular antioxidant, is crucial for keeping the health of periodontal tissues through maintain redox balance and protecting cells from oxidative stress damage. The study of these substances provides potential therapeutic strategies for oral health, and further research will help to reveal their promising applications and mechanisms of action in oral health.

#### 3.2.1 Progress in the application of vitamin C

Vitamin C, as a water-soluble antioxidant, plays a crucial role in oral health, particularly in reducing gingival inflammation. By its antioxidant properties, vitamin C neutralizes free radicals in the oral cavity, thereby reducing oxidative stress and decreasing the release of inflammatory mediators from gingival tissues (Murererehe et al., 2022). Clinical trials have shown that oral or topical application of vitamin C significantly improves gum health, including reducing symptoms of inflammation such as bleeding and swelling (Murererehe et al., 2022). Studies indicate that vitamin C helps promote collagen synthesis and repair, which are crucial for regulating the structure and function of periodontal tissues (Boo YC., 2022). Vitamin C also reduces inflammation-related collagenase activity, further protecting gingival tissues from inflammatory damage. Vitamin C acts by multiple mechanisms, including its antioxidant properties and its regulation of collagen synthesis. Research suggests that vitamin C plays a critical role in regulating collagen synthesis as it is a cofactor for the hydroxylation enzymes necessary for proline and lysine hydroxylation reactions (Naidu, 2017). Additionally, vitamin C enhances immune system function to help keep gum health, involving improving cellular clearance during inflammation and tissue repair processes (Carr and Maggini, 2017). Hence, vitamin C supplementation may contribute to lessen the extent of inflammation which affects gingival tissues through ecreasing collagenase activity, which is vital for keeping gum health.

#### 3.2.2 Research progress on vitamin E

Vitamin E, as a fat-soluble antioxidant, serves as a important protective role in cell membranes through hindering lipid peroxidation, which makes it possibly useful for the prevention and treatment of periodontal health (Sardar et al., 1996). Studies show that vitamin E supplementation can alleviate oxidative pressure levels in periodontal tissues, reduce inflammatory responses, and contribute to stay periodontal health (Lauridsen and Jensen, 2012b). Vitamin E, by its antioxidant properties, neutralizes free radicals and prevents their damage to cell membranes, which is vital for decreasing the occurrence and progression of periodontal diseases like gingivitis (Lauridsen and Jensen, 2012b). Particularly in the treatment of gingivitis and periodontitis, vitamin E may protect periodontal tissues through decreasing the release of inflammatory mediators and other inflammation-related responses (Lauridsen and Jensen, 2012b).

All in all, the mechanisms of action of vitamin E make it a possible therapeutic way for avoiding and treating periodontitis and related periodontal tissue health issues by its antioxidant and anti-inflammatory effects. Further research in this area will help with a better comprehension of the application prospects and mechanisms of vitamin E in oral health.

#### 3.2.3 Study on the role of glutathione

Glutathione (GSH), as the main intracellular antioxidant, is important in maintain the oxidative-reductive balance within cells. Studies have displayed that glutathione is helpful to protect the health of periodontal tissues through decreasing oxidative stress reactions (Ilkun and Boudina, 2013). For instance, some research has indicated that lower levels of glutathione in patients with periodontitis are tightly elated to worsened gum bleeding and deepening of periodontal pockets (Chai and Mieyal, 2023), (Greabu and Calenic, 2013), (Szalai et al., 2009).

A review on the role of glutathione in cellular function emphasizes that glutathione control the oxidative-reductive state within cells through modifying protein S-glutathionylation, influencing multiple cellular signaling pathways and responding to environmental variations inside and outside cells (Chai and Mieyal, 2023). Glutathione’s capacity to maintain oxidative-reductive balance is vital for keeping cells from oxidative damage and maintaining cellular function, which is quite crucial for decreasing the occurrence and progression of periodontal diseases like periodontitis.

Generally, glutathione’s role in cellular function positions it as a possible therapeutic agent for protecting periodontal health by its antioxidant properties and its capacity to maintain oxidative-reductive balance. Further research in this field will develop our insight of glutathione’s application prospects and mechanisms in oral health.

## 4 Clinical research progress

### 4.1 Application advances of antioxidant therapy in human studies

**FIGURE 2:**
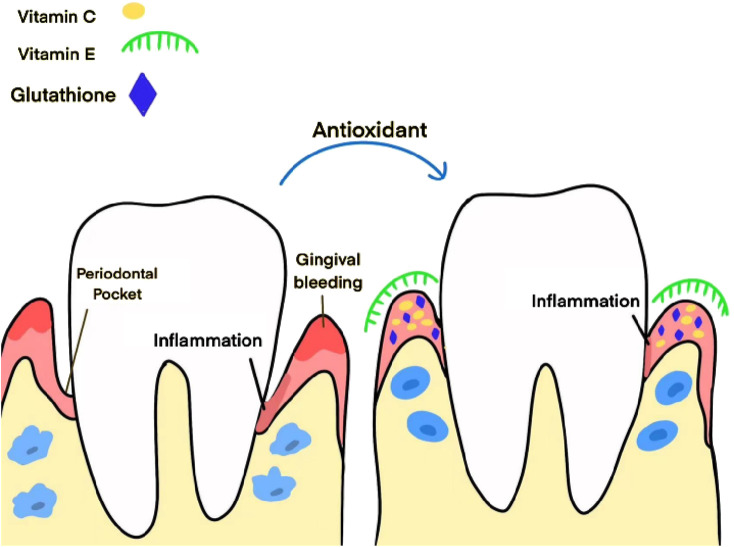
Application advances of antioxidant therapy in human studies. This figure illustrates the mechanism of action of antioxidants on periodontal tissue. This figure on the left shows inflammation around the Periodontal Pocket accompanied by gingival bleeding in the absence of antioxidant intervention. This figure on the right shows that antioxidants such as vitamin C, vitamin E and glutathione, when in their hands, can reduce inflammation in the gum area and improve the health of periodontal tissues, demonstrating the positive role of antioxidants in the maintenance of periodontal health.

Vitamin C and vitamin E, as antioxidants, have displayed imprtant clinical effects in the treatment of periodontitis. Vitamin C, by oral supplementation and local application, reduces symptoms like gingival bleeding and swelling, promoting periodontal tissue health. Vitamin E helps stabilize cell membrane structures, decreases tissue damage resulting from oxidative pressure, and apparently alleviates gingival inflammation and bleeding. Glutathione, as a major intracellular antioxidant, maintains oxidative stress balance, decreases inflammatory responses, and apparently promotes periodontal inflammation indicators, dispalying impvital ortant potential for clinical application. Figure 2 illustrates Application Advances of Antioxidant Therapy in Human Studies.

#### 4.1.1 Clinical trials of vitamin C

Multiple clinical trials have illustrates apparent therapeutic effects of vitamin C as an adjunct in the non-surgical management of periodontitis, both orally and topically. Oral supplementation of vitamin C notably alleviates symptoms like gingival bleeding and swelling, promoting the overall health of periodontal tissues (Fageeh et al., 2021a), (Gomes et al., 2020).

Studies show that vitamin C usefully alleviates clinical symptoms and the risk of disease progression in periodontitis through alleviating oxidative pressure and inflammatory mediator release (Fageeh et al., 2021a), (Gomes et al., 2020). For instance, systematic reviews have indicated that oral vitamin C supplementation as an adjunct to non-surgical periodontitis treatment obviously promotes gingival index, probing depth, and clinical attachment level (Fageeh et al., 2021a).

Besides, local application of vitamin C in the oral environment has also been studied for its antioxidant and anti-inflammatory properties in alleviating the severity of gingival inflammation (Gomes et al., 2020). Hence, vitamin C shows potential not only in oral supplementation but also in local treatments, gradually becoming an vital supplementary method in periodontitis treatment.

#### 4.1.2 Effects of vitamin E application

Vitamin E, as a fat-soluble antioxidant, displays vital advantages following oral supplementation, in particular in the protection and improvement of periodontal tissues (Nagata, 2013). Studies demonstrate that vitamin E stabilizes cell membrane structures, efficiently decreasing periodontal tissue damage resulting from oxidative pressure (Nagata, 2013). For examples, clinical trials have dispalyed that vitamin E supplementation apparently lessens gingival inflammation and bleeding (Hong et al., 2019a).

Moreover, vitamin E contributes to promote the condition of periodontal pockets and lessen gum discomfort and inflammation in patients (Olson and Seidel, 2000a). In a word, vitamin E’s antioxidative properties effciently erase intracellular free radicals, thereby lessening the severity of inflammatory responses. These study results show that vitamin E, following oral supplementation, not only serves as an adjunct therapy to promote symptoms of periodontal diseases but also possibly plays a vital role in keeping periodontal health.

#### 4.1.3 Clinical application of glutathione

As the major intracellular antioxidant, glutathione effciently alleviates oxidative pressure levels in periodontal tissues (Ribas et al., 2014), (Valenti et al., 2023). Studies showthat patients with periodontitis show apparently boosted production of oxygen free radicals, possibly leading to lipid peroxidation, DNA damage, and protein oxidation, worsening tissue damage and inflammatory responses (Fernández-Bravo, 2022), (Varela-López et al., 2018).

Clinical trials show that glutathione supplementation apparently promotes oral health indicators in patients, like lessening periodontal pocket depth and gingival bleeding index. The improvement in these indicators shows glutathione’s potential in alleviating periodontal inflammation and promoting gum healing (Varela-López et al., 2018). Research also shows that glutathione regulates oxidative stress during periodontitis by balancing redox reactions, thereby stopping inflammation development. This mechanism not only applies locally but may also promote oral health by influencing systemic oxidative stress (Varela-López et al., 2018).

All in all, glutathione as a possible therapeutic adjunct in oral disease management displays significant clinical prospects in decreasing oxidative damage and improving oral health. These study results offer reasonable scientific evidence for the application of glutathione in periodontitis treatment.

### 4.2 Comparison and evaluation of different antioxidants

When comparing the roles of vitamin C, vitamin E, and glutathione in the treatment of periodontitis, vitamin C mainly neutralizes water-soluble oxygen free radicals slike hydroxyl radicals and singlet oxygen, protecting the cellular environment and improving the oxidative status of periodontal tissues, resulting into the reduction of inflammation and damage. Vitamin E acts primarily on lipid layers, stabilizing cell membrane structures and avoiding lipid peroxidation damage. Glutathione, as the main intracellular antioxidant, regulates redox reactions and hinders tissue damage lead by oxidative pressure. Clinical trials have demonstrated that both vitamin C and glutathione apparently boost periodontal health indicators like gingival index and periodontal pocket depth, but glutathione provides broader advantages in regulating systemic oxidative stress levels.

#### 4.2.1 Vitamin C vs. vitamin E

When it comes to antioxidative effects, vitamin C primarily neutralizes water-soluble oxygen free radicals, like hydroxyl radicals and singlet oxygen, which protect the cellular environment (Fageeh et al., 2021b), (Tada and Miura, 2019). Literature shows that vitamin C is quite vital in periodontitis treatment through erasing oxygen free radicals and improving cellular oxidative status, thereby reducing inflammation and damage to periodontal tissues (Fageeh et al., 2021b).

Vitamin E, on the other hand, acts mainly on lipid layers, stabilizing cell membrane structures and preventing lipid peroxidation damage. Its antioxidative role primarily concentrates on preventing cell membranes from free radical attacks, thereby keeping the integrity of periodontal tissues (Varela-López et al., 2018).

When it comes to therapeutic effects, both clinical and animal model studies show obvious therapeutic potential of vitamin C and vitamin E in treating periodontitis. Vitamin C is generally used to decrease gingival bleeding and swelling, to improve gingival health (Fageeh et al., 2021b), (Tada and Miura, 2019). Vitamin E helps with lessening periodontal pocket depth and improve clinical attachment level, contributing to thorough periodontal tissue health (Varela-López et al., 2018). When compared to vitamin E, the antioxidant effects of vitamin C are more important in treating periodontal diseases.

#### 4.2.2 Vitamin C vs. glutathione

Comparative research on vitamin C and glutathione shows distinct antioxidative mechanisms and clinical applications in periodontitis treatment. Vitamin C primarily neutralizes water-soluble oxygen free radicals, benefiting to periodontal tissue healing and lessening inflammatory responses (Fageeh et al., 2021b), (Tada and Miura, 2019). By comparision, glutathione functions as the major intracellular antioxidant, which regulates redox reactions and inhibiting oxidative stress-induced tissue damage (Wang et al., 2017b).

Clinical trials show that both vitamin C and glutathione apparently facilitate periodontal health indicators, like gingival index and periodontal pocket depth. Vitamin C reduces gingival bleeding and inflammation, while glutathione boosts the whole antioxidant ability, reducing oxidative stress damage (Wang et al., 2017b; Fageeh et al., 2021b), (Wang et al., 2017b; Fageeh et al., 2021b). Instead, the application of glutathione provides wider benefits through ffecting systemic oxidative pressure levels, possibly promoting overall health beyond periodontal tissues.

To sum up, both vitamin C and glutathione show obvious therapeutic potential in the management of periodontitis. Vitamin C concentrates on neutralizing water-soluble oxygen radicals, while glutathione serves as a systemic antioxidant, managing oxidative stress and enhancing periodontal health.

#### 4.2.3 Vitamin E vs. glutathione

Comparative analysis of vitamin E and glutathione in periodontitis treatment shows obvious antioxidative mechanisms and clinical applications. Vitamin E mainly protects lipid cell membranes, stabilizing cellular structures and preventing lipid peroxidation damage (Varela-López et al., 2018).

On the other hand, glutathione serves as the main intracellular antioxidant, regulating redox reactions and balancing oxidative pressure levels (Wang et al., 2017b). Clinical trials illustrate that both vitamin E and glutathione efficiently promote periodontal health indicators, like gingival index and periodontal pocket depth. Vitamin E lessens gum inflammation and facilitates clinical attachment level, while glutathione boosts comprehensive antioxidant capacity and alleviates oxidative stress-induced tissue damage (Hong et al., 2019b), (Olson and Seidel, 2000b). Nevertheless, glutathione’s application extends beyond periodontal tissues, affecting systemic oxidative pressure levels and probably promoting overall health.

To sum up, both vitamin E and glutathione provide vital therapeutic benefits in managing periodontitis. Vitamin E mainly protects lipid cell membranes, while glutathione serves as an intracellular antioxidant, adjusting redox reactions and systemic oxidative stress levels.

## 5 Potential limitations, challenges and future research directions

### 5.1 Potential limitations and challenges

#### 5.1.1 Dosage issues

The clinical effectiveness of antioxidants often relies on their dose. Doses that are too low may not achieve the desired therapeutic effect, while doses that are too high may lead to side effects. In particular for antioxidants such as vitamin C and vitamin E, high doses can cause gastrointestinal depression or other harmful effects. Moreover, individual differences (e.g., genetic background, metabolic rate, etc.) may influence the absorption and metabolism of antioxidants, leading to different patients responding differently to the same dose. Hence, deciding the proper dosage range and how to regulate the dosage to meet different individuals is a huge challenge in the current application of antioxidant therapy.

#### 5.1.2 Bioavailability

The bioavailability of antioxidants (i.e., the ability of antioxidants to reach target tissues) is one of the key factors in their clinical effectiveness. For instance, vitamin C is less bioavailable when taken orally, and the majoraty of it is absorbed by the intestine and enters the bloodstream, but cannot be totally absorbed by tissues. Others, like glutathione, also have limited bioavailability in oral form because it can be easily degraded during digestion. Hence, how to promote the bioavailability of antioxidants and make them more productive to reach the oral cavity and periodontal tissues is one of the key factors to handling this challenge.

One possible solution is to boost the effective concentration of antioxidants through topical application (e.g., oral spray, local injection, etc.). Nevertheless, such a method also brings problems like dose control, ease of use, and patient compliance, and its feasibility still needs to be further tested in clinical studies.

#### 5.1.3 Long-term effects

The long-term efficiency of antioxidants is also an vital issue in current research. While some studies have demonstrated that antioxidants like vitamins C and E are productive in lessening symptoms of periodontitis and facilitating tissue repair in the short term, data on the consistant effects of their long-term use on periodontal health are limited. Particularly, with long-term use, whether antioxidants are tolerable or whether there are possible side effects, like potential effects on the microbial community, need to be further examined.

Besides, whether the long-term use of antioxidants is useful in stopping the recurrence of periodontitis, as well as the difficulties in remaining efficacy, are also essential aspects to think. In clinical practice, how to secure that patients continue to use antioxidants efficiently during long-term treatment is also an urgent issue to be dealt with.

#### 5.1.4 Individual differences and personalization of treatment

Each patient’s internal environment, immune response, and physiological status are different, which needs us to find a personalized antioxidant treatment plan considering individual differences. Genetic factors, lifestyle factors (e.g., smoking, eating habits), and other complications (e.g., diabetes) can influence the effectiveness of antioxidants. Hence, how to regulate the type, dosage and treatment mode of antioxidants based on the specific situation of the patient to secure the best treatment effect is another main challenge in clinical application.

In short, though antioxidants have emonstrated some possibility in the treatment of periodontitis, their clinical application still has some challenges. Future research needs to apy attention to dose optimization, promoting bioavailability, assessing long-term efficiency, and looking for personalized treatment regimens to make sure the security and productivity of antioxidant therapy.

### 5.2 Future research directions

#### 5.2.1 In-depth exploration of antioxidant therapy mechanisms

Future research can dive deeper and specificlly into the mechanisms of various antioxidants in the treatment of periodontitis. For example, paying attention to the pivotal role of oxidative pressure in the pathogenesis of periodontitis, further research can focus on how antioxidants decrease the production of reactive oxygen species or boost the body’s antioxidant defense systems to promote the inflammatory status and fix capacity of periodontal tissues. Moreover, understanding the specific molecular mechanisms of antioxidants which interact with inflammatory mediators and immune cells can offer a more integrited insight of the pathways of antioxidant therapy’s efficacy.

#### 5.2.2 Improvements in clinical trial design and interpretation of results

In improvement of clinical trial designs, considerations could involve conducting multicenter trials, enhancing randomization and double-blind designs, and utilizing more comprehensive outcome evaluation metrics like clinical symptom improvement, changes in inflammatory markers, and periodontal tissue regeneration. Besides, to better interpret trial results, systematic review and meta-analysis methods could be introduced to thoroughly assess outcomes from different trials, providing more reliable evidence for the clinical implementation of antioxidant therapies.

#### 5.2.3 Potential applications of antioxidants in personalized therapy

For possible applications in personalized therapy, future research can combine genomics and biomarker studies to explore individual patient responses to specific antioxidants. When analyzing patients' genetic backgrounds, environmental factors, and physiological statuses can benefit to personalize treatment plans, boosting therapeutic efficacy and prognosis management. What’s more, using information technologies such as artificial intelligence and big data analysis can enable personalized analysis of large-scale data and optimization of treatment strategies, laying the foundation for accurate applications of antioxidant therapies in managing periodontitis.

#### 5.2.4 The role of lifestyle factors in the regulation of oxidative stress

In addition to antioxidants, lifestyle factors (e.g., diet, smoking, etc.) are also important in the onset and progression of periodontitis. Exploring the moderating role of these factors in oxidative pressure is not only useful to understand the mechanism of periodontitis, but also offers tailered intervention approaches for clinical practice. Foods high in sugar, fat, and low in antioxidants in the modern diet may boost oxidative pressure and improve the development of periodontitis. Otherwise, a diet rich in antioxidants, like vitamins C, E and carotenoids, can contribute to alleviate inflammation and improve periodontal health. Excessive intake of processed foods, high-sugar drinks and trans fats may boost the progression of periodontitis through promoting the oxidative pressure response. Cigarette smoking is a obvious risk factor for periodontitis, exacerbating oxidative pressure and makes periodontal tissue more susceptible to damage through boosting free radical generation and hindering the activity of the antioxidant enzyme system. Smoking not only contributes to gum inflammation, but is also likely to further worsen periodontitis through stimulating the immune system and affecting the microbial community. Hence, decreasing alcohol consumption should be considered in lifestyle interventions. Given that lifestyle factors and antioxidant application can contribute to develop a more thorough treatment plan, in particular in tailered treatment design, which can more usefully regulate the occurrence and progression of periodontitis.

#### 5.2.5 Biotechnological advances and future prospects for periodontitis treatment

Nanomedicine and CRISPR gene editing technology offer a brand-new idea for the treatment of periodontitis. Though these technologies are still in the research stage, they display huge potential for precision therapy and tissue repair. Through encapsulating drugs, antioxidants, and biomolecules in nanoparticles, nanotechnology enables accurate targeting of diseased areas, promoting drug bioavailability and decreasing side effects. Studies have illustrated that nanoparticles can efficiently penetrate diseased tissues, carry drugs or antioxidants, alleviate oxidative pressure and inflammation, and boost periodontal tissue repair. Nanoparticles can also be applied to develop intelligent delivery systems releasing drugs under specific conditions, therby boosting therapeutic efficacy. CRISPR/Cas9 gene editing technology can be applied to fix gene mutations linked with periodontal disease, control immune responses, and repair the normal function of periodontal tissues. For instance, using CRISPR to repair genes affecting periodontal health may help reduce inflammation and facilitate tissue repair capabilities. Furthermore, CRISPR can also modulate the periodontal microbiota, optimize the host-microbial relationship, and promote the therapeutic effect. Despite their promise, these technologies still have technical and ethical challenges. The biocompatibility, targeting, and release mechanisms of nanoparticles need to be further studied, and the safety and efficacy of CRISPR technology in periodontal therapy, especially in avoiding genetic variation, need to be further explored. Future research should concentrate on the clinical translation of the technology and handle safety, long-term efficacy, and ethical issues. In summary, nanomedicine and CRISPR gene editing technology are expected to hugely facilitate the treatment effect of periodontitis through precision therapy and gene repair, and become an vital tool for personalized treatment.

#### 5.2.6 Biomarkers progression

With develpoments in genomics, molecular biology, and biomarker research, tailored treatment has demostrated great potential in the management of periodontitis. Future research should concentrate on how to utilize patients' genomic information and biomarkers to design more precise treatment options. For example, by identifying genetic susceptibility genes linked with periodontitis, integrited with specific microbial community characteristics, personalized intervention strategies can be designed to more usefully suppress the course of the disease and facilitate tissue repair. Nowadays, though the relevant clinical cases have not yet been popularized, with the maturity of genomics technology and the accumulation of data, the practice of personalized treatment will gradually be applied into clinical practice. We look forward to providing scientific basis and clinical validation for personalized treatment of periodontitis by the mix of multi-center clinical trials and precision medicine in the future.

## 6 Conclusion

### 6.1 Current status and future prospects of antioxidant therapy in periodontitis

Current research shows that antioxidant therapy has significant potential in the management of periodontitis. Through decreasing reactive oxygen species generation and boosting antioxidant defense systems, antioxidants usefully improve the inflammatory status of periodontal tissues, facilitating tissue repair and regeneration. Despite positive research findings, antioxidant therapy still faces challenges in clinical applications, like consistency in treatment outcomes and long-term durability of effects. Looking forward, with more research into antioxidant therapy mechanisms and personalized applications, wider applications in managing periodontitis can be expected, offering more useful treatment options for patients.

### 6.2 Key focus areas and challenges in future research

Key focus areas for future research should focus on: first of all, exploring in-depth the mechanisms of action of different antioxidants and their advantages and applicability in periodontitis treatment; besides, promoting clinical trial designs to improve the scientific validity and clinical utility of trial results; and finally, diving into personalized treatment strategies through combining genomics and biomarker research for precise patient treatments. Challenges comprise of standardized assessment of treatment outcomes, antioxidant selection and dosage issues, and ongoing observation and evaluation of long-term effects. Through addressing these challenges, wider prospects can be opened for the application of antioxidant therapy in the management of periodontitis.
